# Recruitment via the Internet and Social Networking Sites: The 1989-1995 Cohort of the Australian Longitudinal Study on Women’s Health

**DOI:** 10.2196/jmir.3788

**Published:** 2014-12-15

**Authors:** Gita Devi Mishra, Richard Hockey, Jennifer Powers, Deborah Loxton, Leigh Tooth, Ingrid Rowlands, Julie Byles, Annette Dobson

**Affiliations:** ^1^Centre for Longitudinal and Life Course ResearchSchool of Public HealthUniversity of QueenslandHerstonAustralia; ^2^Priority Research Centre for Gender, Health and AgeingUniversity of NewcastleNewcastleAustralia

**Keywords:** Web-based survey, online survey, social media, Facebook, representativeness, education, socioeconomic factors, women’s health, data collection, young adults, Australia

## Abstract

**Background:**

Faced with the challenge of recruiting young adults for health studies, researchers have increasingly turned to the Internet and social networking sites, such as Facebook, as part of their recruitment strategy. As yet, few large-scale studies are available that report on the characteristics and representativeness of the sample obtained from such recruitment methods.

**Objective:**

The intent of the study was to describe the sociodemographic and health characteristics of a national sample of young Australian women recruited mainly through the Internet and social networking sites and to discuss the representativeness of their sociodemographic, health, and lifestyle characteristics relative to the population.

**Methods:**

A cohort of 17,069 women (born between 1989 and 1995) was recruited in 2012-13 for the Australian Longitudinal Study on Women’s Health. Sociodemographic characteristics (percentages, means, and 95% confidence intervals) from the online survey data were compared with women aged 18-23 years from the 2011 Australian Census. Sample data were compared by age and education level with data from the 2011-13 Australian Health Survey (AHS).

**Results:**

Compared to the Australian Census data, study participants were broadly representative in terms of geographical distribution across Australia, marital status (95.62%, 16,321/17,069) were never married), and age distribution. A higher percentage had attained university (22.52%, 3844/17,069) and trade/certificate/diploma qualifications (25.94%, 4428/17,069) compared with this age group of women in the national population (9.4% and 21.7% respectively). Among study participants, 22.05% (3721/16,877) were not in paid employment with 35.18% (5931/16,857) studying 16 or more hours a week. A higher percentage of study participants rated their health in the online survey as fair or poor (rather than good, very good, or excellent) compared with those participating in face-to-face interviews in the AHS (18.77%, 3203/17,069 vs 10.1%). A higher percentage of study participants were current smokers (21.78%, 3718/17,069 vs 16.4%) and physically active (59.30%, 10,089/17,014 were classified as sufficiently active vs 48.3%) but alcohol consumption was lower (59.58%, 9865/16,558 reported drinking alcohol at least once per month vs 65.9% in the AHS). Using self-reported height and weight to determine body mass index (BMI, kg/m^2^), 34.80% (5901/16,956) of the cohort were classified as overweight or obese (BMI of 25 or more), compared with 33.6% respectively using measured height and weight in the AHS.

**Conclusions:**

Findings indicated that using the Internet and social networking sites for an online survey represent a feasible recruitment strategy for a national cohort of young women and result in a broadly representative sample of the Australian population.

## Introduction

Recruitment of participants for longitudinal health studies poses increasing challenges for researchers, with indications of declining participation rates in telephone- or mail-based data collection surveys [1-3]. Recruitment and retention are particularly acute issues with respect to young adults, who are highly mobile and difficult to reach by conventional modes of contact, such as landline telephone or postal address [4,5]. Young adults’ familiarity with digital and mobile technologies, however, provides an opportunity for innovative recruitment and survey modalities including the Internet and social networks. Although recent research suggests that participant recruitment via social networking sites has advantages over traditional methods (eg, convenient, cost effective, reaches young adults), it is often described as introducing a participant self-selection bias, limiting the representativeness of the sample [6]. The issue of sample representativeness is the subject of ongoing debate but its relevance requires consideration of the research questions and study design [7-14].  Representativeness may not be important, or even desirable, for etiological studies, if the measurement and control of confounders is feasible [9,12]. However, representativeness is important if one of the goals of the study is to estimate the prevalence of disease or health status of population groups [11,13]. Also, having a sample of sufficient size and diversity in terms of a range of key characteristics and exposures is an essential attribute for many epidemiological studies [3,8,15]. It remains unclear, however, if recruitment strategies that use the Internet and social networks are able to obtain a representative sample of young adults for large national cohort studies.

Given the logistics and financial challenges of using conventional survey methods, increasingly the Internet, in conjunction with integrated database management systems, has been used to access a large sampling frame of potential participants [16-18]. Yet a distinction should be drawn between relying on social network sites, such as Facebook, for recruitment and the separate implementation of an online survey where a variety of recruitment methods direct participants to the survey website. The latter is exemplified by the recent French NutriNet-Santé study where television advertisements provided the major strategy for recruitment, achieving over 88,000 participants, but promotion of the study via the Internet and radio also contributed substantially [19]. Some large established cohort studies, such as the Black Women’s Health Study [20], have also changed their data collection methods to online surveys for the follow-up surveys. The Australian Longitudinal Study on Women’s Health (ALSWH), which includes three age cohorts, has transferred the two younger cohorts, one born in 1973-1978, the other born in 1946-1951 (originally recruited in 1996), from postal questionnaires to online surveys (with postal paper surveys available, if requested) [21].

While online or Web-based questionnaires can assist with survey completion, they do not specifically address the issue of recruiting a representative sample of young adults. The increased access to the Internet by young adults and the concomitant rise in popularity of online social networks has provided a way forward for health researchers. Recent surveys in the United States have found that 76% and 83% of 18-29 year olds have access to broadband or a smartphone respectively [22,23], and 73% of adults who go online use a social networking site of some kind [24]. Facebook has emerged as the preeminent social networking platform, with an estimated 1.2 billion monthly users and monthly usage statistics indicating 12 million unique Australian visitors [25]. Overall, young Australian adults display similar Internet usage patterns to those evident in the United States [26], with 92% of 18 to 24 year olds visiting social networking sites on a regular basis [27], most (95%) of whom are Facebook members [28].

Social networks can be defined as groups of people with some shared pattern of contacts or interactions between them [29]. Researchers have used Facebook advertising to target people with specific health conditions and lifestyles [4,26,30-33], but these studies have typically been small scale [4,31-33] or rely on an identified attribute that characterizes the network to facilitate a snowball recruitment strategy (eg, peer referral to the study) [34]. The reliance on social connections may be challenging for national health surveys that seek a representative or comprehensive sample of the population [33] and—importantly for the Australian context—a sufficient sample of young adults from rural and remote areas. However, Facebook offers a convenient, immediate, and low-cost way to contact a broad sample of eligible young adults and targets advertising dynamically to specific sociodemographic groups that are under-represented among study participants.

The paper reports on a large national sample of young Australian women (aged 18 to 23 years) and who were primarily recruited through Facebook advertising and other Internet-based modes of contact. We compare the sociodemographic, health, and lifestyle characteristics of this cohort with women in the same age range from the 2011 Australian Census and the 2011-12 Australian Health Survey (AHS).

## Methods

### Study Design

Since its baseline survey in 1996 of over 40,000 Australian women, the ALSWH has become established as the Australia-wide study of women’s health, with surveys conducted approximately every 3 years since 1998 [35]. Until recently, the study comprised three cohorts of women born in 1921-26, 1946-51, and 1973-78. These women were randomly selected using the national health insurance database (Medicare), which includes all permanent residents of Australia. Comparison of demographic characteristics of participants at baseline with census data indicated that the samples are broadly representative of the Australian population in these age groups [36].

This paper uses data collected from a new young ALSWH cohort of 17,069 women born from 1989-95 and recruited in 2012-13. Women will be surveyed annually with the primary aim of identifying changes in health and well-being and health service needs across the lifespan, to inform Australian policies across a range of issues. Eligible women were those aged 18-23 years when they completed the surveys, who had a valid Medicare number (this includes all permanent residents, but not temporary residents, such as overseas students). The women also needed to consent to having their survey data linked with administrative health data on their health service utilization. Approval for the study was obtained from the Human Research Ethics Committee of the University of Newcastle and the University of Queensland, as well as the Department of Human Services and the Department of Health. Further details of the survey methodology are available from the study website [21].

###  Recruitment

Initially we planned to recruit the new cohort of young women using the same methodology adopted for the previous cohorts, with contact by mail, however, this approach was reassessed when a pilot survey using these methods for another Australian study with women of a similar age yielded only a 6% response rate [5]. Subsequently, an array of recruitment strategies was deployed: advertising through Facebook or other online media (eg, study website, Gumtree, Twitter, Instagram, Tumblr, YouTube), referral (word-of-mouth by study staff members and their networks, professional bodies, and participants who had already completed the survey), and conventional media advertising (eg, posters, flyers, magazines, TV, and radio interviews). Cinema advertising was also tried in some regional areas. Over the recruitment period, two distinct campaigns were conducted. The first was designed by study staff members and offered the chance to win one of 100 AU $50 gift vouchers (October 2012-September 2013) and the second was coordinated by a marketing company and offered the chance to win one of 2000 exclusive pairs of leggings designed by an independent clothing designer (October 2013-December 2013). Resources progressively shifted from conventional media to online social media according to the observed response rates. Of all the methods adopted, targeted advertising through Facebook was the most successful means of recruitment (69.94%, 11,799/16,869), followed by the marketing company campaign (12.72%, 2145/16,869), referral (7.02% 1184/16,869), conventional media (5.39%, 910/16,869), and other online media (4.93%, 831/16,869).

Data were collected via a Web-based survey. Eligibility was assessed by asking participants to submit their personal and contact details. Eligible study participants were asked 62 questions on: sociodemographic and personal characteristics (eg, educational qualifications), aspects of physical and mental health (eg, self-rated general health), anthropometric data (height, weight), health risk factors (eg, physical activity levels), risk-taking behavior (eg, illicit drug use), access to health service use (eg, screening services), reproductive health and outcomes (eg, pregnancy, birth outcomes), and experience of violence or abuse. Survey features, such as organizing the questions by topic, limiting the number of questions to only one or two per page, using a multiple choice format where possible, and a visible progress bar were used to encourage survey completion and to minimize participant burden.

Demographic data from the study participants were routinely compared with 2011 Census data. The Australian Bureau of Statistics conducts the Australian Census every 5 years, with the most recent being on 9 August 2011. The Census measures key sociodemographic characteristics of all people who are in Australia on Census Night, including their education level and marital status [37]. Advertising strategies were then dynamically adapted according to the areas or demographic groups identified as being under-represented in the sample as it accrued. The recruitment period for the cohort ran for 14 months from 26 October 2012 to 19 December 2013.

Characteristics of the study participants were also compared with women in the same age group from the 2011-13 Australian Health Survey (AHS), a large national health survey. Initially, 30,721 households were approached and of these, 25,080 (81.64%) responded, resulting in 31,837 participants. Face-to-face interviews with one adult from each household collected data on a range of health-related issues, including health status, risk factors, socioeconomic circumstances, physical activity, and nutrition [38]. The height and weight of participants, used for the BMI classification, were obtained from measured rather than self-reported data.

### Sociodemographic, Health-Related, and Lifestyle Characteristics

Data for sociodemographic variables were re-categorized to facilitate comparison with the 2011 Australian Census data: age (in years); State/Territory of residence; area of residence based on an index of distance to the nearest urban center (major cities, inner regional, outer regional, remote, very remote) [39]; education level completed (less than year 12, year 12 or equivalent, certificate/diploma, university degree); Aboriginal or Torres Strait Island origin (no, yes); and current relationship status (never married, married, separated/divorced/widowed).

Similarly, data for health-related and lifestyle variables were recoded to enable comparison with the 2011-2013 AHS, as follows: self-rated health (excellent, very good, good, fair or poor); smoking status (non-smoker, current smoker); body weight (kg); height (cm); body mass index (BMI); underweight (<18.5 kg/m^2^); normal weight (18.5 to <25 kg/m^2^); overweight (25 to <30 kg/m^2^); obese (≥30 kg/m^2^), according to the World Health Organization’s classification [40]; and alcohol consumption (never drink, less than once a month, less than once a week, at least once a week) [41]. A physical activity category was derived from questions on the frequency and duration of different types of physical activity (inactive, insufficiently active, sufficiently active) [42].

### Statistical Analysis

The sociodemographic characteristics of the sample (percentages, means, and 95% confidence interval) were compared with corresponding data from women in the same age group in the 2011 Census. The prevalence of health-related and lifestyle characteristics were then compared with the 2011-2013 AHS. Based on preliminary analysis, and to enable comparison with the AHS data, weights for the sample, W(x), at each education level x, were calculated as:

W(x) = (N/P) × (P(x)/N(x))

where N is the number of women in the sample and N(x) is the number of women in the sample with education level x. Similarly, P is the number of women in the 18-23 year age group in the Australian population and P(x) is the number of women in the 18-23 year age-group in the Australian population with education level x. Women who had missing data for their education level (7.8%) were omitted from the calculation of weights, which in effect assumes that the data are missing at random. The unweighted and the weighted data are presented.

## Results

### Sociodemographic Characteristics

Comparison with the 2011 Census data (Table 1) indicates that the study participants were broadly representative in their geographical distribution across Australia (both in terms of State or Territory and area of residence): three-quarters of young women (75.28%, 12,849/17,069) resided in major cities, compared to 74.5% among the population. Similarly to young women in the Census, the vast majority (95.62%, 16,321/17,069) had never been married. The age distribution of the study participants was also close to that of the population.

The main difference identified was that study participants had higher levels of educational attainment, for instance, only 7.45% (1271/17,069) had not completed year 12 (compared with 14.9% of the women in population in this age group). Slightly more than one-third of women (35.18%, 5931/16,857) were studying 16 or more hours a week (Table 2).

**Figure 1:**
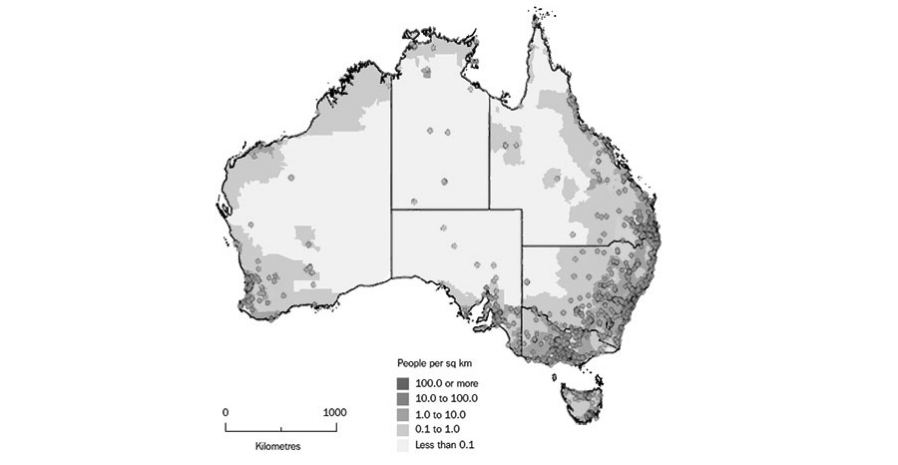
Distribution of women aged 18-23 years recruited using the Internet across Australia (N=17,069). Each dot represents at least 1 woman. Figure adapted from the Regional Population Growth, Australia (catalogue 3218.0.).

Figure 1 illustrates the broad geographical distribution of the cohort (with each dot representing at least one individual) and reflects the relatively high population density along the East and South coast and the sparse population scattered across the central and northwest areas of the continent.

**Table 1:** Comparison of sociodemographic characteristics of women aged 18-23 years, recruited using the Internet, with 2011 Australian Census data.

Characteristic		Study participants, 2012-2013 (N=17,069)		Census data, 2011 (N=844,636)
		n^a^ (%)	95% CI	%
**Age**				
	18	2599 (15.2)	14.7-15.8	16.0
	19	2986 (17.5)	17.0-18.1	16.2
	20	2924 (17.1)	16.6-17.7	16.8
	21	2809 (16.5)	15.9-17.0	17.1
	22	2879 (16.9)	16.3-17.4	16.9
	23	2851 (16.7)	16.2-17.3	17.0
**State/Territories**				
	New South Wales	4741 (27.9)	27.2-28.5	31.0
	Victoria	4089 (24.0)	23.4-24.7	25.4
	Queensland	3807 (22.4)	21.8-23.0	20.6
	Western Australia	1883 (11.1)	10.6-11.5	10.6
	South Australia	1301 (7.6)	7.2-8.0	7.3
	Australian Capital Territory	560 (3.3)	3.0-3.6	2.0
	Tasmania	494 (2.9)	2.7-3.2	2.1
	Northern Territory	138 (0.8)	0.7-0.9	1.0
**Area of residence**				
	Major city	12,849 (75.3)	74.6-75.9	74.5
	Inner regional	2831 (16.6)	16.0-17.1	16.0
	Outer regional	1151 (6.7)	6.4-7.1	7.2
	Remote	131 (0.8)	0.6-0.9	1.1
	Very remote	52 (0.3)	0.2-0.4	0.9
	Migratory/no usual address/missing	55 (0.3)	0.2-0.4	0.3
**Highest qualification**				
	Less than Year 12	1271 (7.4)	7.1-7.9	14.9
	Year 12 or equivalent	7341 (43.0)	42.8-44.2	46.1
	Trade/certificate / diploma	4428 (26.0)	25.6-26.9	21.7
	University degree	3844 (22.5)	21.1-23.4	9.4
	Missing/not stated/ inadequately described	185 (1.2)	1.0-1.3	7.8
**Aboriginal or Torres Strait Islander origin** ^b^				
	No	14,529 (97.4)	97.2-97.7	91.9
	Yes	384 (2.6)	2.3-2.8	3.5
	Missing	-	-	4.7
**Marital status**				
	Never married	16,321 (95.6)	95.2-95.9	94.5
	Married	510 (3.0)	2.7-3.2	4.9
	Separated/divorced/widowed	53 (0.3)	0.2-0.4	0.6
	Missing	185 (1.2)	1.10-1.3	-

^a^Numbers may not sum to total due to missing data.

^b^N=14,913 as this question was only asked in a later version of the survey.

**Table 2:** Comparison of education level and lifestyle characteristics of women aged 18-23 years, recruited using the Internet and social networking sites, with the 2011-2012 Australian National Health Survey.

		Study participants 2012-2013 (N=17,069)		Study participants (weighted for education level)	Australian National Health Survey 2011-2013
		n^a^ (%)	95% CI	% (95% CI)	%
**Education level**					
	Less than Year 12	1271 (7.4)	7.1-7.9	15.4 (14.6-16.1)	17.9
	Year 12 or equivalent	7341 (43.0)	42.8-44.2	49.7 (48.9-50.5)	40.1
	Certificate/diploma	4428 (26.0)	25.6-26.9	23.4 (22.8-24.1)	32.9
	University degree	3844 (22.5)	21.1-23.4	10.4 (10.0-10.7)	8.7
	Missing/not stated /inadequ descr	185 (1.2)	1.0-1.3	1.1 (1.0-1.3)	0.4
**Self-rated health (weighted)**					
	Excellent	1097 (6.5)	6.1-6.9	5.8 (5.5-6.2)	17.7
	Very good	6081 (36.0)	35.3-36.7	33.8 (33.0-34.5)	38.1
	Good	6866 (40.6)	39.9-41.4	41.7 (40.9-42.5)	34.4
	Fair/poor	2859 (16.9)	16.3-17.5	18.8 (18.1-19.4)	10.1
**Smoking status**					
	Non-smoker	13,701 (81.1)	80.5-81.7	78.3 (77.6-79.0)	85.0
	Current smoker	3188 (18.9)	18.3-19.5	21.8 (21.0-22.4)	16.4
**Body mass index (kg/m^2^; weighted)**					
	Underweight (<18.5)	1332 (7.9)	7.5-8.3	7.8 (7.4-8.3)	5.4
	Normal weight (18.5-25)	9923 (59.1)	58.3-59.8	57.4 (56.6-58.2)	58.2
	Overweight (25-30)	3231 (19.2)	18.7-19.9	19.5 (18.9-20.2)	14.8
	Obese (>30)	2298 (13.7)	13.2-14.2	15.3 (14.6-15.9)	18.8
Mean weight (kg)		67.4	67.1-67.6	67.8 (67.5-68.0)	66.4
Mean height (cm)		166.2	166.1-166.4	166.1 (166.0-166.2)	164.6
Mean BMI		24.4	24.3-24.5	24.6 (24.5-24.7)	24.7
**Physical activity level**					
	Inactive	1024 (6.0)	5.6-6.4	6.7 (6.3-7.2)	9.8
	Insufficiently active	5631 (33.1)	32.4-33.8	34.0 (33.2-34.8)	41.3
	Sufficiently active	10,359 (60.9)	60.2-61.6	59.3 (58.5-60.1)	48.3
**Alcohol consumption (weighted)**					
	Never drink	731 (4.5)	4.2-4.8	5.0 (4.6-5.3)	12.7
	Less than once a month	5629 (34.4)	33.7-35.2	35.5 (34.7-36.3)	21.4
	Less than once a week	5572 (34.1)	33.2-34.8	33.6 (32.8-34.4)	31.9
	At least once a week	4417 (27.0)	26.3-27.7	26.0 (25.2-26.7)	34.0
**Paid employment**					
	Yes	13,156 (77.9)	77.3-78.6	74.9 (74.2-75.7)	-
	No	3721 (22.1)	21.4-22.7	25.1 (24.3-25.8)	-
**Studying (≥16 hrs per week)**					
	Yes	5931 (35.2)	34.5-35.9	33.6 (32.8-34.3)	-
	No	10,926 (64.8)	64.1-65.5	66.4 (65.7-67.2)	-

^a^Numbers may not sum to total due to missing data.

### Health-Related and Lifestyle Characteristics

A higher percentage of study participants rated their health in the online survey as fair or poor than women participating in face-to-face interviews in the AHS (18.77%, 3203/17,069 from weighted data vs 10.1% respectively) (Table 2). Study participants reported higher rates of smoking (21.78%, 3718/17,069 vs 16.4% were current smokers) and physical activity (58.97%, 10,150/17,211 were classified as sufficiently active vs 48.3% in the AHS) but lower levels of alcohol consumption than women in the AHS (59.58%, 9865/16,558 vs 65.9% drank alcohol at least once per month). Based on self-reported height and weight, 19.54% (3313/16,956) of the participants were classified as overweight (25≤ BMI< 30), 15.26% (2588/16,956) were obese (BMI≥30), compared with 14.8% and 18.8% respectively in the AHS.

The percentages obtained from unweighted data were similar to the education-weighted data and did not make substantive changes in the comparisons with AHS data.

## Discussion

### Principal Results

This study examines the representativeness, in terms of sociodemographic and lifestyle characteristics of a national cohort of young Australian women (born in 1989-95) who were recruited in 2012-13 mainly via social media and other Internet platforms, and completed the baseline survey online. The area of residence of the study participants is broadly representative of the geographical distribution of the population. The main sociodemographic difference was the higher proportion of women who had post-secondary school qualifications. The percentages for current smokers and those who were physically active among study participants were higher than the findings from the AHS. Based on self-reported height and weight, more than one-third of young women were identified as overweight or obese, similar to the percentage in these combined BMI categories found in the AHS. However, within these categories there were differences between studies: a higher percentage of study participants was overweight than in the AHS and a lower percentage was obese.

These comparisons used data from study participants weighted to match the education level of women in this age group in the national population. Little substantive difference in the distribution of health and lifestyle characteristics is evident when using unweighted data from study participants.

### Limitations

There are a number of considerations to take into account when comparing prevalence estimates across population-based studies. For example, the eligibility criterion of the ALSWH limits study participants to those with the Medicare number, whereas the Census data are based the entire population including visitors, and the AHS survey was a sample of those living in Australia for at least 1 year or with the intention of doing so. Thus, unlike ALSWH, both the Census and AHS data included the sizable number of women students from overseas studying in Australia [43]. It is also possible that some of the women may have based their educational level on their current studies (for a university degree or trade qualification) rather than their completed qualifications. Over-representation of participants with higher educational levels is also recognized as a characteristic of many epidemiological studies [4,35], including the previously recruited cohorts in ALSWH. Also, AHS has a sample size of women in the similar age group of about 1000, considerably smaller than the number of study participants in this age group in ALSWH.

Another issue concerns differences in the mode of survey administration. For instance, the AHS was conducted via a face-to-face interview [43], whereas the study participants completed an online questionnaire. This difference may have a varying degree of impact according to the nature of the survey question and social or cultural factors that may influence an open response among some women, such as for levels of alcohol consumption. Furthermore, variations in the wording of questions or the available response options, such as for physical activity level, may limit the comparability of results. Yet it is worth noting that even though the BMI data for the study participants were calculated from self-assessed height and weight, whereas for the AHS height and weight were measured directly, the proportion of those classified as overweight or obese were similar in both studies. This is consistent with a previous study that found that Web-based self-reported data provide a valid measure of weight [44].

While 2.57% (384/14,913) of the study participants identified themselves as Aboriginal or Torres Strait Islander women, this is lower than the 3.5% from the 2011 Census data. This was expected, since effective recruitment and retention of participants from the Indigenous population requires culturally specific protocols that are best implemented in a separate and specially designed study.

### Comparison With Prior Work

Previous small scale studies have used online social networking sites for recruitment but this is one of the few, population-based studies to rely on dynamically targeted advertising through Facebook to recruit a large cohort of young women for a national longitudinal health study. Other studies have also examined the cost-effectiveness of Facebook for recruitment [4,26,45], but only one study by Fenner et al [4] reported on the characteristics of the subsequent sample. Although for a smaller scale study, Fenner et al [4] also targeted young Australian women via Facebook, using separate advertising campaigns to target different age groups and regions, and were successful in obtaining a broadly representative of young women.

### Implications

The representativeness of the sample in terms of key attributes, such as sociodemographic characteristics, is necessary to maximize external validity and strengthen the evidence base for policy and health care planning [46]. It is central to describing the health of a population at a particular point in time.

It is not the only consideration, however, as sufficient size and heterogeneity of the sample are important attributes for research on the relationships between risk factors and health outcomes and patterns over time that can provide insights on the underlying causal mechanisms at work. Size and diversity of the sample are also important for identifying the health status and health care needs of minority groups.

### Conclusions

Findings from this study support the use of the Internet and social networking sites as a viable recruitment method for large heterogeneous samples of young adults who are broadly representative of the population. Researchers need to be mindful that given the rapidly changing landscape of online social media, the exact strategies likely to be most effective for recruitment may also vary over time and according to the targeted subpopulation or age group of interest.

## Abbreviations

AHS: Australian Health Survey
ALSWH: Australian Longitudinal Study on Women’s Health
BMI: body mass index
